# Supplementary material for: Unveiling the Bioactive Potential of the Invasive Jellyfish Phyllorhiza punctata Through Integrative Transcriptomic and Proteomic Analyses
Source: Biomolecules. 2025 Aug 4;15(8):1121. doi: 10.3390/biom15081121 (PMC12383608; doi:10.3390/biom15081121)
Supplement: Supplementary file 1 [file biomolecules-15-01121-s001.zip › Suplementary Material/FileS3/PEPFOLD4/XP_065070585_15270/clusters.html]

 PEP-FOLD clusters


Download archive of all models  here   

|  |  |  |
| --- | --- | --- |
| # | file | sOPEP |
| # cluster 1 - Size: 2 |  |  |
|  | XP\_065070585\_15270-model1 | -18.3219 |
|  | XP\_065070585\_15270-model1.1 | -18.1841 |
| # cluster 2 - Size: 5 |  |  |
|  | XP\_065070585\_15270-model2 | -17.9132 |
|  | XP\_065070585\_15270-model2.1 | -17.7302 |
|  | XP\_065070585\_15270-model2.2 | -17.6564 |
|  | XP\_065070585\_15270-model2.3 | -17.5921 |
|  | XP\_065070585\_15270-model2.4 | -17.5736 |
| # cluster 3 - Size: 1 |  |  |
|  | XP\_065070585\_15270-model3 | -16.4732 |
| # cluster 4 - Size: 3 |  |  |
|  | XP\_065070585\_15270-model4 | -14.8957 |
|  | XP\_065070585\_15270-model4.1 | -14.0223 |
|  | XP\_065070585\_15270-model4.2 | -13.4683 |
| # cluster 5 - Size: 2 |  |  |
|  | XP\_065070585\_15270-model5 | -14.8106 |
|  | XP\_065070585\_15270-model5.1 | -13.6717 |
| # cluster 6 - Size: 1 |  |  |
|  | XP\_065070585\_15270-model6 | -13.9745 |
| # cluster 7 - Size: 11 |  |  |
|  | XP\_065070585\_15270-model7 | -13.9353 |
|  | XP\_065070585\_15270-model7.1 | -12.0112 |
|  | XP\_065070585\_15270-model7.2 | -11.8728 |
|  | XP\_065070585\_15270-model7.3 | -11.8407 |
|  | XP\_065070585\_15270-model7.4 | -11.8044 |
|  | XP\_065070585\_15270-model7.5 | -11.7746 |
|  | XP\_065070585\_15270-model7.6 | -11.7659 |
|  | XP\_065070585\_15270-model7.7 | -11.5906 |
|  | XP\_065070585\_15270-model7.8 | -11.5694 |
|  | XP\_065070585\_15270-model7.9 | -11.378 |
|  | XP\_065070585\_15270-model7.10 | -11.2237 |
| # cluster 8 - Size: 1 |  |  |
|  | XP\_065070585\_15270-model8 | -13.2558 |
| # cluster 9 - Size: 5 |  |  |
|  | XP\_065070585\_15270-model9 | -13.1355 |
|  | XP\_065070585\_15270-model9.1 | -13.0296 |
|  | XP\_065070585\_15270-model9.2 | -12.9868 |
|  | XP\_065070585\_15270-model9.3 | -12.6237 |
|  | XP\_065070585\_15270-model9.4 | -10.7533 |
| # cluster 10 - Size: 7 |  |  |
|  | XP\_065070585\_15270-model10 | -13.1223 |
|  | XP\_065070585\_15270-model10.1 | -12.7302 |
|  | XP\_065070585\_15270-model10.2 | -11.9217 |
|  | XP\_065070585\_15270-model10.3 | -11.7775 |
|  | XP\_065070585\_15270-model10.4 | -11.5936 |
|  | XP\_065070585\_15270-model10.5 | -11.378 |
|  | XP\_065070585\_15270-model10.6 | -11.1862 |
| # cluster 11 - Size: 5 |  |  |
|  | XP\_065070585\_15270-model11 | -12.7571 |
|  | XP\_065070585\_15270-model11.1 | -12.5364 |
|  | XP\_065070585\_15270-model11.2 | -12.2344 |
|  | XP\_065070585\_15270-model11.3 | -11.5439 |
|  | XP\_065070585\_15270-model11.4 | -11.4461 |
| # cluster 12 - Size: 3 |  |  |
|  | XP\_065070585\_15270-model12 | -12.2148 |
|  | XP\_065070585\_15270-model12.1 | -10.6212 |
|  | XP\_065070585\_15270-model12.2 | -10.5925 |
| # cluster 13 - Size: 3 |  |  |
|  | XP\_065070585\_15270-model13 | -12.1655 |
|  | XP\_065070585\_15270-model13.1 | -11.9015 |
|  | XP\_065070585\_15270-model13.2 | -11.5258 |
| # cluster 14 - Size: 2 |  |  |
|  | XP\_065070585\_15270-model14 | -12.1276 |
|  | XP\_065070585\_15270-model14.1 | -11.6375 |
| # cluster 15 - Size: 5 |  |  |
|  | XP\_065070585\_15270-model15 | -12.032 |
|  | XP\_065070585\_15270-model15.1 | -11.885 |
|  | XP\_065070585\_15270-model15.2 | -10.8291 |
|  | XP\_065070585\_15270-model15.3 | -10.319 |
|  | XP\_065070585\_15270-model15.4 | -10.2537 |
| # cluster 16 - Size: 17 |  |  |
|  | XP\_065070585\_15270-model16 | -12.0316 |
|  | XP\_065070585\_15270-model16.1 | -10.6311 |
|  | XP\_065070585\_15270-model16.2 | -10.6022 |
|  | XP\_065070585\_15270-model16.3 | -10.5652 |
|  | XP\_065070585\_15270-model16.4 | -10.5619 |
|  | XP\_065070585\_15270-model16.5 | -10.5077 |
|  | XP\_065070585\_15270-model16.6 | -10.4769 |
|  | XP\_065070585\_15270-model16.7 | -10.4469 |
|  | XP\_065070585\_15270-model16.8 | -10.2989 |
|  | XP\_065070585\_15270-model16.9 | -10.296 |
|  | XP\_065070585\_15270-model16.10 | -10.2937 |
|  | XP\_065070585\_15270-model16.11 | -10.2862 |
|  | XP\_065070585\_15270-model16.12 | -10.2389 |
|  | XP\_065070585\_15270-model16.13 | -10.0316 |
|  | XP\_065070585\_15270-model16.14 | -9.96972 |
|  | XP\_065070585\_15270-model16.15 | -9.91363 |
|  | XP\_065070585\_15270-model16.16 | -9.90656 |
| # cluster 17 - Size: 1 |  |  |
|  | XP\_065070585\_15270-model17 | -11.9946 |
| # cluster 18 - Size: 3 |  |  |
|  | XP\_065070585\_15270-model18 | -11.6284 |
|  | XP\_065070585\_15270-model18.1 | -11.5526 |
|  | XP\_065070585\_15270-model18.2 | -11.3977 |
| # cluster 19 - Size: 3 |  |  |
|  | XP\_065070585\_15270-model19 | -11.4325 |
|  | XP\_065070585\_15270-model19.1 | -11.2665 |
|  | XP\_065070585\_15270-model19.2 | -11.0571 |
| # cluster 20 - Size: 2 |  |  |
|  | XP\_065070585\_15270-model20 | -11.3556 |
|  | XP\_065070585\_15270-model20.1 | -11.3299 |
| # cluster 21 - Size: 3 |  |  |
|  | XP\_065070585\_15270-model21 | -11.2097 |
|  | XP\_065070585\_15270-model21.1 | -10.7434 |
|  | XP\_065070585\_15270-model21.2 | -10.4378 |
| # cluster 22 - Size: 2 |  |  |
|  | XP\_065070585\_15270-model22 | -10.9881 |
|  | XP\_065070585\_15270-model22.1 | -10.4094 |
| # cluster 23 - Size: 2 |  |  |
|  | XP\_065070585\_15270-model23 | -10.9748 |
|  | XP\_065070585\_15270-model23.1 | -10.8183 |
| # cluster 24 - Size: 1 |  |  |
|  | XP\_065070585\_15270-model24 | -10.9509 |
| # cluster 25 - Size: 3 |  |  |
|  | XP\_065070585\_15270-model25 | -10.848 |
|  | XP\_065070585\_15270-model25.1 | -10.8325 |
|  | XP\_065070585\_15270-model25.2 | -10.1001 |
| # cluster 26 - Size: 1 |  |  |
|  | XP\_065070585\_15270-model26 | -10.7488 |
| # cluster 27 - Size: 1 |  |  |
|  | XP\_065070585\_15270-model27 | -10.6612 |
| # cluster 28 - Size: 1 |  |  |
|  | XP\_065070585\_15270-model28 | -10.5377 |
| # cluster 29 - Size: 2 |  |  |
|  | XP\_065070585\_15270-model29 | -10.523 |
|  | XP\_065070585\_15270-model29.1 | -10.2402 |
| # cluster 30 - Size: 1 |  |  |
|  | XP\_065070585\_15270-model30 | -10.3653 |
| # cluster 31 - Size: 1 |  |  |
|  | XP\_065070585\_15270-model31 | -10.353 |
|  |  |  |
